# Draft Genome Sequences of Pseudomonas sp. Strains MWU 12-2029, MWU 12-3088, and MWU 12-3091, Isolated from Wild and Cultivated Massachusetts Cranberry Bogs

**DOI:** 10.1128/mra.00889-22

**Published:** 2022-10-17

**Authors:** Akhila Manthena, Arin Pittman, Scott Soby

**Affiliations:** a Arizona College of Osteopathic Medicine, Midwestern University, Glendale, Arizona, USA; b Biomedical Sciences, College of Graduate Studies, and College of Veterinary Medicine, Midwestern University, Glendale, Arizona, USA; Loyola University Chicago

## Abstract

Pseudomonas sp. strains MWU12-2029, MWU12-3088, and MWU12-3091 were isolated from wild and cultivated cranberry bog soils in southeastern Massachusetts. The three isolates are closely related to Pseudomonas kribbensis, a not validly published member of the P. fluorescens group, and contain three putative insecticidal protein genes, including the toxin complex A gene (*tcaC*).

## ANNOUNCEMENT

Pseudomonas spp. comprise a considerable component of bacteria isolated from cultivated and wild cranberry bogs during a multiyear culture-dependent survey conducted at the Cape Cod National Seashore (MWU12-2029) (42.070624N, 70.210548W) and the University of Massachusetts State Bog (MWU12-3088 and MWU12-3091) (41.766767N, 70.66842W) (1–8). Although presumably an important constituent of these wetland microbiomes, little is known about how these organisms affect the dynamics of the bog ecosystem, including their effects on insects. As an initial foray into understanding the cranberry bog soil microbiome, 5 cm by 5 cm soil samples were taken from cultivated and wild bogs in July 2012 for isolating and characterizing bacterial populations. Approximately 1 g from soil cores was vortexed in sterile water, and the rinsate was plated onto King’s medium B (KMB) agar supplemented with 50 μg mL^−1^ each of cycloheximide and ampicillin and incubated at 26°C for 48 h. Fluorescent colonies were purified three times on KMB and stored at −80°C in 34% glycerol. Isolates from frozen storage were recovered on KMB agar, and populations were inoculated into KMB broth cultures grown overnight for genomic DNA (gDNA) isolation. All kits described below were used according to the manufacturers’ instructions. gDNA was extracted with a DNeasy blood and tissue kit (Qiagen, USA), and Kapa Biosystems Hyperplus library preparation kits (catalog number KK8514; Roche, USA) were used to generate Illumina-compatible genomic DNA libraries: DNA was enzymatically sheared to ~500 bp, end repaired, and A tailed; Illumina-compatible adapters with unique indexes (catalog number 00989130v2; IDT, Coralville, IA) were then ligated to each sample; and adapter-ligated molecules were cleaned using Kapa pure beads (catalog number KK8002) and amplified with Kapa Hifi enzyme (catalog number KK2502). Library fragment sizes were determined on an Agilent TapeStation system and quantified by quantitative PCR (qPCR) (Kapa library quantification kit, catalog number KK4835) on a QuantStudio 5 system (Thermo Fisher, USA). Samples were multiplex pooled and sequenced on an Illumina MiSeq platform in a 2-by-250 flow cell. The software was set to default settings except as indicated below. Raw reads were assembled with Unicycler v0.4.8 (9) and polished with Pilon v1.23 (10) within the PATRIC v3.6.12 comprehensive genome analysis pipeline, except for the trim setting, which was set to “true” (11). The comprehensive analysis pipeline includes quality control and trimming by QUAST v5.0.2 (12) and Trim Galore v0.4.0 (13) and annotation by RASTtk v1.073 (14), supplemented with antiSMASH v6.0 (15) for the recognition of secondary metabolite gene clusters. Using the Type (Strain) Genome Server (TYGS), isolates were placed with high confidence within the genus Pseudomonas (16). All three isolates were most closely related to Pseudomonas kribbensis 46-2^T^ (GenBank accession number CP029608) but fell below the 70% digital DNA-DNA hybridization (dDDH_d4_) (TYGS v342) (16–18) or 95 to 96% average nucleotide identity by BLAST analysis (ANIb) (JSpeciesWS v3.9.5) (19, 20) cutoff to be included in that species (Table 1).

**TABLE 1:** Genomic data summary

Isolate	BioSample accession no.	GenBank accession no.	SRA accession no.	Genome size (bp)	No. of contigs	*N*_50_ (bp)	G+C content (%)	Mean read length (bp)	No. of reads	Coverage (×)	dDDH_d4_/ANIb with *P. kribbensis* 46-2^T^ (%)
MWU12-2029	SAMN26814158	JALJDY000000000	SRR18644892	6,318,446	38	583,019	60.68	233.75	3,403,188	126	65.1/95.48
MWU12-3088	SAMN26803934	JALJEA000000000	SRR18645887	6,305,590	37	392,904	60.67	229.72	3,774,982	138	65.6/95.65
MWU12-3091	SAMN26896969	JALJET000000000	SRR18508983	6,305,411	38	392,833	60.67	224.57	3,190,686	114	65.6/95.64

MWU12-2029, MWU12-3088, and MWU12-3091 all contain presumptive genes for insecticidal toxin complex A (*tcaC*) (21) and two additional putative insecticidal toxins that are widespread among members of the Pseudomonas fluorescens group.

### Data availability.

The whole-genome shotgun sequencing project has been deposited in the DDBJ/EMBL/GenBank database under BioProject accession number PRJNA691338 with the accession numbers in Table 1. The versions described in this paper are GenBank accession numbers JALJDY000000000.1 (MWU12-2029), JALJEA000000000.1 (MWU12-3088), and JALJET000000000.1 (MWU12-3091). RASTtk annotations are available under an open license at Zenodo (see https://zenodo.org/record/6413129#.YwaiEUfMKUk, https://zenodo.org/record/6413119#.Ywah6kfMKUk, and https://zenodo.org/record/6413231#.Ywain0fMKUk).
